# 2-Chloro-1,2-diphenyl­ethanone (desyl chloride)

**DOI:** 10.1107/S1600536811014541

**Published:** 2011-04-22

**Authors:** Richard Betz, Cedric McCleland, Eric Hosten

**Affiliations:** a Nelson Mandela Metropolitan University, Summerstrand Campus, Department of Chemistry, University Way, Summerstrand, PO Box 77000, Port Elizabeth 6031, South Africa

## Abstract

The title compound, C_14_H_11_ClO, is a racemic derivative of benzoin. Its carbonyl group adopts a nearly eclipsed conformation with the Cl substituent characterized by a dihedral angle of 17.5 (2)°. The closest intermolecular π–π contact is 4.258 (1) Å.

## Related literature

For the crystal structure of benzoin, see: Haisa *et al.* (1980 ▶); Sole *et al.* (1998 ▶). For the crystal structure of 2-phenyl­acetophenone, see: Rieker *et al.* (1993 ▶). For the crystal structure of 2-chloro­acetophenone, see: Grossert *et al.* (1984 ▶). Structures containing similar angles were retrieved from the Cambridge Structural Database (Allen, 2002 ▶).
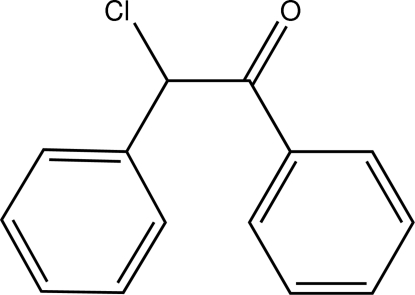


## Experimental

### 

#### Crystal data

C_14_H_11_ClO
*M* *_r_* = 230.68
Monoclinic, 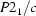

*a* = 12.6233 (11) Å
*b* = 5.8227 (5) Å
*c* = 15.6745 (14) Å
β = 97.317 (3)°
*V* = 1142.72 (17) Å^3^
*Z* = 4
Mo *K*α radiation
μ = 0.31 mm^−1^
*T* = 200 K
0.53 × 0.29 × 0.16 mm

#### Data collection

Bruker APEXII CCD diffractometer
9777 measured reflections
2816 independent reflections
2366 reflections with *I* > 2σ(*I*)
*R*_int_ = 0.024

#### Refinement

*R*[*F* ^2^ > 2σ(*F* ^2^)] = 0.037
*wR*(*F* ^2^) = 0.104
*S* = 1.07
2816 reflections
145 parameters
H-atom parameters constrained
Δρ_max_ = 0.36 e Å^−3^
Δρ_min_ = −0.42 e Å^−3^

### 

Data collection: *APEX2* (Bruker, 2010 ▶); cell refinement: *SAINT* (Bruker, 2010 ▶); data reduction: *SAINT*; program(s) used to solve structure: *SHELXS97* (Sheldrick, 2008 ▶); program(s) used to refine structure: *SHELXL97* (Sheldrick, 2008 ▶); molecular graphics: *ORTEP-3* (Farrugia, 1997 ▶) and *Mercury* (Macrae *et al.*, 2006 ▶); software used to prepare material for publication: *SHELXL97* and *PLATON* (Spek, 2009 ▶).

## Supplementary Material

Crystal structure: contains datablocks I, global. DOI: 10.1107/S1600536811014541/ld2009sup1.cif

Supplementary material file. DOI: 10.1107/S1600536811014541/ld2009Isup2.cdx

Structure factors: contains datablocks I. DOI: 10.1107/S1600536811014541/ld2009Isup3.hkl

Additional supplementary materials:  crystallographic information; 3D view; checkCIF report

## Figures and Tables

**Fig. 1.**
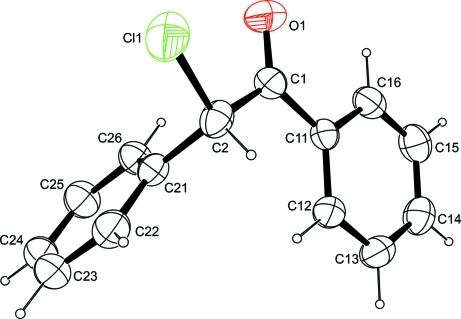
The molecular structure of the title compound, with atom labels and anisotropic displacement ellipsoids drawn at 50% probability level.

**Fig. 2.**
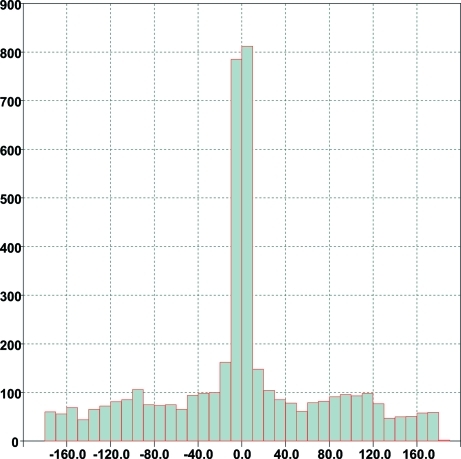
Statistical distribution of O=C—C—Cl dihedral angles (data based on CSD search including all deposited crystal structures up to November 2010).

## supplementary crystallographic information

### Comment

The title compound was studied as a reference structure for a series of transition-metal complexes employing it as a ligand.

Bond lengths and angles are usual. The torsion angle O=C—C—Cl is 17.5 (2) °. A statistics of values for the similar angles reported in the CSD (Allen, 2002) shows that this eclipsed conformation is the most preferable for α-cloroketones (Fig. 1 and Fig. 2). However, possibly due to steric hindrances from the bulky phenyl group next to the Cl substituent, the dihedral value is somewhat distorted in comparison to the molecular structure of 2-chloroacetophenone (Grossert *et al.* (1984)), where the respective angle was found at 2.4 °.

Unlike the crystal structure of 2-chloroacetophenone, which is dominated by strong C–H···O and C–H···Cl contacts, the crystal structure of the title compound does not show any intermolecular contacts whose range falls short of the sum of van-der-Waals radii. The closest π···π-contact was measured at 4.258 (1) Å.

### Experimental

The compound was synthesized by reacting benzoin with thionyl chloride. Crystals suitable for X-ray diffraction were obtained upon recrystallization from ethanol.

### Refinement

Carbon-bound H-atoms were placed in calculated positions (C—H 0.99 Å for the methylene group and C—H 0.95 Å for aromatic carbon atoms) and were included in the refinement in the riding model approximation, with *U*(H) set to 1.2*U*_eq_(C).

### Crystal data

**Table:**

C_14_H_11_ClO	*F*(000) = 480
*M**_r_* = 230.68	*D*_x_ = 1.341 Mg m^−^^3^
Monoclinic, *P*2_1_/*c*	Mo *K*α radiation, λ = 0.71073 Å
Hall symbol: -P 2ybc	Cell parameters from 5815 reflections
*a* = 12.6233 (11) Å	θ = 2.6–28.3°
*b* = 5.8227 (5) Å	µ = 0.31 mm^−^^1^
*c* = 15.6745 (14) Å	*T* = 200 K
β = 97.317 (3)°	Rod, colourless
*V* = 1142.72 (17) Å^3^	0.53 × 0.29 × 0.16 mm
*Z* = 4	

### Data collection

**Table:**

Bruker APEXII CCD diffractometer	2366 reflections with *I* > 2σ(*I*)
Radiation source: fine-focus sealed tube	*R*_int_ = 0.024
graphite	θ_max_ = 28.3°, θ_min_ = 2.6°
φ and ω scans	*h* = −16→16
9777 measured reflections	*k* = −7→6
2816 independent reflections	*l* = −20→20

### Refinement

**Table:**

Refinement on *F*^2^	Primary atom site location: structure-invariant direct methods
Least-squares matrix: full	Secondary atom site location: difference Fourier map
*R*[*F*^2^ > 2σ(*F*^2^)] = 0.037	Hydrogen site location: inferred from neighbouring sites
*wR*(*F*^2^) = 0.104	H-atom parameters constrained
*S* = 1.07	*w* = 1/[σ^2^(*F*_o_^2^) + (0.0471*P*)^2^ + 0.3595*P*] where *P* = (*F*_o_^2^ + 2*F*_c_^2^)/3
2816 reflections	(Δ/σ)_max_ < 0.001
145 parameters	Δρ_max_ = 0.36 e Å^−^^3^
0 restraints	Δρ_min_ = −0.42 e Å^−^^3^

### Fractional atomic coordinates and isotropic or equivalent isotropic displacement parameters (Å^2^)

**Table:**

	*x*	*y*	*z*	*U*_iso_*/*U*_eq_	
Cl1	0.36760 (3)	0.54577 (9)	0.02422 (2)	0.05873 (16)	
O1	0.19610 (9)	0.8528 (2)	0.05035 (8)	0.0585 (3)	
C1	0.17721 (11)	0.6644 (2)	0.07711 (8)	0.0369 (3)	
C2	0.26188 (10)	0.4753 (2)	0.08663 (8)	0.0361 (3)	
H2	0.2277	0.3291	0.0637	0.043*	
C11	0.07080 (10)	0.6085 (2)	0.10402 (7)	0.0318 (3)	
C12	0.04806 (11)	0.4010 (2)	0.14191 (9)	0.0369 (3)	
H12	0.1014	0.2853	0.1511	0.044*	
C13	−0.05239 (11)	0.3628 (3)	0.16633 (10)	0.0436 (3)	
H13	−0.0673	0.2221	0.1931	0.052*	
C14	−0.13073 (11)	0.5290 (3)	0.15188 (10)	0.0454 (3)	
H14	−0.1996	0.5017	0.1682	0.054*	
C15	−0.10909 (12)	0.7350 (3)	0.11366 (10)	0.0456 (3)	
H15	−0.1632	0.8486	0.1032	0.055*	
C16	−0.00869 (11)	0.7752 (2)	0.09067 (8)	0.0395 (3)	
H16	0.0063	0.9181	0.0655	0.047*	
C21	0.30932 (9)	0.4355 (2)	0.17883 (8)	0.0313 (3)	
C22	0.36130 (11)	0.2294 (2)	0.20111 (10)	0.0411 (3)	
H22	0.3639	0.1128	0.1590	0.049*	
C23	0.40932 (12)	0.1938 (3)	0.28445 (11)	0.0485 (4)	
H23	0.4461	0.0542	0.2991	0.058*	
C24	0.40391 (12)	0.3604 (3)	0.34634 (10)	0.0483 (4)	
H24	0.4364	0.3348	0.4036	0.058*	
C25	0.35139 (12)	0.5639 (3)	0.32497 (9)	0.0451 (3)	
H25	0.3470	0.6780	0.3677	0.054*	
C26	0.30485 (11)	0.6021 (2)	0.24116 (8)	0.0372 (3)	
H26	0.2697	0.7436	0.2265	0.045*	

### Atomic displacement parameters (Å^2^)

**Table:**

	*U*^11^	*U*^22^	*U*^33^	*U*^12^	*U*^13^	*U*^23^
Cl1	0.0447 (2)	0.0974 (4)	0.0365 (2)	−0.0071 (2)	0.01475 (15)	−0.00083 (19)
O1	0.0538 (6)	0.0513 (7)	0.0703 (8)	−0.0085 (5)	0.0077 (6)	0.0241 (6)
C1	0.0385 (6)	0.0400 (7)	0.0312 (6)	−0.0051 (5)	0.0003 (5)	0.0047 (5)
C2	0.0319 (6)	0.0449 (7)	0.0321 (6)	−0.0058 (5)	0.0068 (5)	−0.0049 (5)
C11	0.0332 (6)	0.0338 (6)	0.0271 (5)	−0.0019 (5)	−0.0011 (4)	0.0001 (5)
C12	0.0344 (6)	0.0325 (6)	0.0429 (7)	−0.0001 (5)	0.0020 (5)	0.0033 (5)
C13	0.0412 (7)	0.0392 (7)	0.0508 (8)	−0.0069 (6)	0.0075 (6)	0.0037 (6)
C14	0.0335 (7)	0.0558 (9)	0.0472 (8)	−0.0025 (6)	0.0066 (6)	−0.0046 (7)
C15	0.0403 (7)	0.0479 (8)	0.0474 (8)	0.0116 (6)	0.0010 (6)	−0.0026 (6)
C16	0.0459 (7)	0.0355 (7)	0.0358 (7)	0.0032 (6)	0.0003 (5)	0.0031 (5)
C21	0.0268 (5)	0.0337 (6)	0.0339 (6)	−0.0021 (5)	0.0063 (4)	−0.0003 (5)
C22	0.0364 (7)	0.0336 (7)	0.0539 (8)	0.0008 (5)	0.0081 (6)	−0.0030 (6)
C23	0.0386 (7)	0.0413 (8)	0.0646 (10)	0.0038 (6)	0.0026 (6)	0.0153 (7)
C24	0.0422 (7)	0.0584 (9)	0.0423 (7)	−0.0050 (7)	−0.0018 (6)	0.0155 (7)
C25	0.0503 (8)	0.0510 (9)	0.0335 (7)	−0.0008 (7)	0.0029 (6)	−0.0026 (6)
C26	0.0413 (7)	0.0348 (7)	0.0351 (6)	0.0041 (5)	0.0031 (5)	−0.0002 (5)

### Geometric parameters (Å, °)

**Table:**

Cl1—C2	1.7997 (13)	C15—C16	1.381 (2)
O1—C1	1.2092 (18)	C15—H15	0.9500
C1—C11	1.4941 (18)	C16—H16	0.9500
C1—C2	1.529 (2)	C21—C26	1.3829 (18)
C2—C21	1.5100 (18)	C21—C22	1.3910 (18)
C2—H2	1.0000	C22—C23	1.384 (2)
C11—C12	1.3921 (18)	C22—H22	0.9500
C11—C16	1.3929 (18)	C23—C24	1.379 (2)
C12—C13	1.3883 (19)	C23—H23	0.9500
C12—H12	0.9500	C24—C25	1.378 (2)
C13—C14	1.381 (2)	C24—H24	0.9500
C13—H13	0.9500	C25—C26	1.3871 (19)
C14—C15	1.384 (2)	C25—H25	0.9500
C14—H14	0.9500	C26—H26	0.9500
			
O1—C1—C11	121.31 (13)	C14—C15—H15	120.1
O1—C1—C2	121.41 (13)	C15—C16—C11	120.67 (13)
C11—C1—C2	117.27 (11)	C15—C16—H16	119.7
C21—C2—C1	113.02 (10)	C11—C16—H16	119.7
C21—C2—Cl1	108.91 (9)	C26—C21—C22	119.22 (12)
C1—C2—Cl1	109.90 (10)	C26—C21—C2	121.45 (12)
C21—C2—H2	108.3	C22—C21—C2	119.30 (12)
C1—C2—H2	108.3	C23—C22—C21	120.13 (13)
Cl1—C2—H2	108.3	C23—C22—H22	119.9
C12—C11—C16	119.03 (12)	C21—C22—H22	119.9
C12—C11—C1	123.44 (12)	C24—C23—C22	120.25 (14)
C16—C11—C1	117.52 (12)	C24—C23—H23	119.9
C13—C12—C11	120.11 (13)	C22—C23—H23	119.9
C13—C12—H12	119.9	C25—C24—C23	119.92 (14)
C11—C12—H12	119.9	C25—C24—H24	120.0
C14—C13—C12	120.18 (14)	C23—C24—H24	120.0
C14—C13—H13	119.9	C24—C25—C26	120.06 (14)
C12—C13—H13	119.9	C24—C25—H25	120.0
C13—C14—C15	120.10 (13)	C26—C25—H25	120.0
C13—C14—H14	120.0	C21—C26—C25	120.40 (13)
C15—C14—H14	120.0	C21—C26—H26	119.8
C16—C15—C14	119.89 (13)	C25—C26—H26	119.8
C16—C15—H15	120.1		
			
O1—C1—C2—C21	104.41 (15)	C12—C11—C16—C15	−0.8 (2)
C11—C1—C2—C21	−74.29 (14)	C1—C11—C16—C15	179.73 (12)
O1—C1—C2—Cl1	−17.47 (17)	C1—C2—C21—C26	−21.38 (17)
C11—C1—C2—Cl1	163.83 (9)	Cl1—C2—C21—C26	101.06 (13)
O1—C1—C11—C12	−174.40 (14)	C1—C2—C21—C22	160.45 (11)
C2—C1—C11—C12	4.30 (18)	Cl1—C2—C21—C22	−77.11 (13)
O1—C1—C11—C16	5.09 (19)	C26—C21—C22—C23	−1.02 (19)
C2—C1—C11—C16	−176.21 (11)	C2—C21—C22—C23	177.18 (12)
C16—C11—C12—C13	−0.4 (2)	C21—C22—C23—C24	1.4 (2)
C1—C11—C12—C13	179.07 (12)	C22—C23—C24—C25	−0.6 (2)
C11—C12—C13—C14	1.1 (2)	C23—C24—C25—C26	−0.6 (2)
C12—C13—C14—C15	−0.6 (2)	C22—C21—C26—C25	−0.2 (2)
C13—C14—C15—C16	−0.6 (2)	C2—C21—C26—C25	−178.35 (13)
C14—C15—C16—C11	1.3 (2)	C24—C25—C26—C21	1.0 (2)
